# Prognosis of renal re-transplantation for chronic graft failure

**DOI:** 10.3389/fsurg.2026.1826503

**Published:** 2026-05-26

**Authors:** Xueyu Gong, Yuewen Liu, Fuhai Hui, Naiyue Shi, Boqian Wang, Xueyi Wang, Yijian Zhang, Hongwei Yang, Long He, Yan Zhang

**Affiliations:** 1 Department of Organ Transplant Center, General Hospital of Northern Theater Command, Shenyang, Liaoning, China; 2 Shenyang Pharmaceutical University, Shenyang, Liaoning, China

**Keywords:** prognosis, renal re-transplantation, retrospective study, survival rate of kidney transplantation, survival rate of transplanted kidney

## Abstract

**Objective:**

To analyze the clinical characteristics, patient survival rate, and graft survival rate differences between renal re-transplantation recipients (ReTR) and first-time recipients (FTR) of matched donor kidneys by comparing clinical data, providing evidence for improving graft survival rates in re-transplantation patients.

**Methods:**

A retrospective analysis was conducted on renal transplant patients from the Organ Transplant Center of the Northern Theater Command General Hospital between 2020 and 2024. The patients were divided into two groups: the recipient group (ReTR) who received a second kidney transplant and the first recipient group (FTR) who received a matched donor kidney. Clinical data from both groups were collected, and postoperative follow-up was performed.

**Results:**

The group of recipients undergoing renal re-transplantation and the group of initial recipients with matched donor kidneys were each 24 cases. Compared with the initial recipients with matched donor kidneys, the recipients of renal re-transplantation exhibited higher positive rate of population reactive antibodies (PRA) (*P* = 0.0145) preoperatively, lower platelet and serum albumin levels at 7 days postoperatively, with statistically significant differences (*P* = 0.006; *P* = 0.046), and a significant increase in uric acid levels at 14 days postoperatively (*P* = 0.021); no significant differences were observed in other indicators. There was no significant difference in the incidence of delayed graft function (DGF) between the two groups (45.8% vs. 50%, *P* = 0.773). Survival analysis results indicated that the graft survival rate of patients undergoing renal re-transplantation was lower than that of patients receiving initial transplantation, with a statistically significant difference (*P* = 0.022), but no significant difference in survival rates (*P* = 0.281).Multivariate COX regression analysis results indicated that hypoalbuminemia at 7 days postoperatively was an independent risk factor for mortality in patients after transplantation (HR: 2.387,95.0% CI: 1.072–5.319, *P* = 0.033). Secondary transplantation (HR: 81.867, 95.0% CI: 1.164–5,758.747, *P* = 0.042) and postoperative DGF (HR: 156.163, 95.0% CI: 1.249–19,531.98, *P* = 0.04) were independent risk factors for graft failure.

**Conclusions:**

The graft survival period in patients undergoing repeat renal transplantation is lower than that in first-time renal transplantation, but there is no significant difference in overall survival. Attention should be paid to prevention of postoperative DGF occurrence.Other clinical indicators such as platelet changes, and patients nutritional status should be improved, with a low-purine diet as the mainstay, and uric acid-lowering drugs should be used when necessary.

## 1 Introduction

Renal transplantation is an effective treatment for end-stage renal disease (ESRD). The increasing survival rate of patients, combined with relatively static improvements in long-term graft outcomes, has led to a growing number of transplant recipients facing graft failure, ultimately resulting in the choice of dialysis or retransplantation (1). However, some patients require repeat kidney transplantation (re-KT) due to renal failure caused by various factors. Re-KT is the preferred treatment option for patients with graft failure (2). For patients with a history of failed kidney transplantation, repeat kidney transplantation (re-KT) is a superior therapeutic option compared to dialysis, reducing the 5-year mortality risk by 23%–45% (3). For patients with transplant failure, repeat renal transplantation demonstrates superior survival outcomes and quality of life compared to returning to dialysis therapy (4, 5).

However, compared to primary kidney transplantation, patients undergoing repeat kidney transplantation face more complex clinical conditions, with a significantly increased risk of sensitization during the procedure (6). The incidence of rejection reactions is significantly increased, and the surgical difficulty is also higher (7). Currently, there is relatively limited research on patients undergoing repeat kidney transplantation. This study conducted a follow-up study on patients who underwent repeat transplantation with the initial recipients with matched donor kidneys. Utilizing matched donor kidneys effectively eliminates donor-related confounding factors, including donor characteristics, kidney quality, and immunogenic background, thereby markedly enhancing the internal validity and reliability of research outcomes in renal transplantation.The findings aim to provide evidence for improving graft survival rates in repeat transplant recipients, which holds significant implications for developing rational treatment strategies and enhancing patient outcomes in clinical practice.

## 2 Materials and methods

This study conducted a comprehensive clinical data collection and follow-up of patients undergoing repeat kidney transplantation and first-time kidney transplantation from the same donor at the Organ Transplantation Center of the Northern Theater Command General Hospital from January 2020 to December 2024. It analyzed factors influencing the prognosis of repeat kidney transplantation patients, including basic clinical characteristics and transplantation-related factors, to accurately assess the outcomes of repeat kidney transplantation.

### 2.1 General information

A total of 48 renal transplantation cases from January 2020 to December 2024 were included in the study, comprising 24 recipients of repeat renal transplantation and 24 primary recipients of matched donor kidneys, with a 1:1 patient-to-donor ratio. The maximum HLA mismatch among donors was 4.

1. Inclusion criteria for the retransplantation group
  1. Age 18 years or older.
  2. The cause of initial transplant failure is clearly identified (e.g., rejection reaction, graft failure, vascular complications, etc.)
  3. The interval between re-transplantation ≥3 months
  4. The patients medical records are complete, including detailed preoperative, intraoperative, and postoperative information.
2. Inclusion criteria for the first transplantation group
  1. Age 18 years or older.
  2. Patients with renal transplantation indications and first-time transplantation for renal failure
  3. The patients medical records are complete, including detailed preoperative, intraoperative, and postoperative information.
3. Exclusion Criteria
  1. Patients with other severe systemic diseases, such as malignant tumors or serious cardiovascular and cerebrovascular diseases, which may significantly affect the prognosis assessment.
  2. recipients of multi-organ combined transplantation;
  3. Patients in pregnancy or lactation;
  4. Patients with severe data deficiencies that preclude valid analysis.

### 2.2 Data collection

1. Basic clinical information of the patient, including age, gender, height, weight, body mass index (BMI), cause of uremia, comorbidities, duration of dialysis, systolic blood pressure, diastolic blood pressure, heart rate, smoking history, time since last transplantation, postoperative complications, and population reactivity antibody (PRA). Preoperative use of anti-rejection drugs in patients undergoing repeat transplantation.
2. Laboratory parameters: Including preoperative and postoperative complete blood count (CBC) (white blood cell count, neutrophil-to-lymphocyte ratio, platelet count, hemoglobin), renal function indicators (serum creatinine, serum urea nitrogen, serum cystatin), cardiac function indicators (Creatine Kinase MB, CK-MB), N-terminal pro-brain natriuretic peptide (NT-proBNP), myoglobin), hepatic function indicators (alanine aminotransferase (ALT), aspartate aminotransferase (AST), total bilirubin, alkaline phosphatase), albumin, electrolytes (sodium, potassium, phosphorus), metabolic parameters (blood glucose, uric acid, triglycerides, low-density lipoprotein), lymphocyte subpopulation analysis, etc.
3. Drug therapy regimen: Preoperative anti-rejection induction regimen (ATG, basiliximab), postoperative anti-rejection drugs include tacrolimus/cyclosporine, glucocorticoids, and enteric-coated mycophenolate mofetil/mycophenolate mofetil.

### 2.3 Outcome measures

1. Primary outcomes: Overall patient survival rate; graft survival time (OPTN/UNOS defined as no need for restart of dialysis therapy or subsequent renal transplantation)
2. Secondary outcome: Delayed graft function recovery (defined as serum creatinine >400 μmol/L in the first week post-renal transplantation or requiring hemodialysis treatment) (8).

### 2.4 Statistical methods

Data analysis was performed using IBM SPSS Statistics 27. Measurement data were expressed as mean ± standard deviation, with intergroup comparisons analyzed by independent samples *t*-test; count data were presented as frequency (%), analyzed by chi-square test; survival analysis was conducted using Kaplan–Meier method and Log Rank test. Analysis of risk factors for transplant renal failure and recipient mortality were used COX regression analysis. A *P*-value <0.05 was considered statistically significant.

## 3 Results

### 3.1 The general conditions of the recipients for ReTR and FTR group

The mean age of the first renal transplant recipients was 48.5 ± 10.7 years, while that of the repeat renal transplant recipients was 46.88 ± 10.5 years. No significant differences were observed between the two groups in terms of gender, BMI, blood pressure, heart rate, smoking history, comorbidities, or dialysis duration. The preoperative positive rate of PRA was significantly higher in repeat renal transplant recipients compared to first renal transplant recipients (54.2% vs. 16.7%, *P* = 0.0145) (see Table 1).

**Table 1:** Comparison of general conditions between FTR and ReTR groups.

Characteristics	FTR group (*n* = 24)	ReTR group (*n* = 24)	*P value*
Age (Year)	46.88 ± 10.5	48.54 ± 10.71	0.589
Male	19 (79.2%)	14 (58.3%)	0.119
Female	5 (20.8%)	10 (41.7%)	
BMI (kg/m²)	22.41 ± 4.63	22.95 ± 4.34	0.681
SBP (mmHg)	147.92 ± 21.08	143.54 ± 23.54	0.501
DBP (mmHg)	91.83 ± 12.63	87.17 ± 9.71	0.158
Heart rate	88.08 ± 14.61	87.92 ± 12.97	0.967
Smoking history	5 (20.8%)	0	0.267
Complications			
Diabetes	2 (8.3%)	4 (16.7%)	0.383
Coronary heart disease	1 (4.2%)	2 (8.3%)	0.551
Preoperative PRA positive	4 (16.7%)	13 (54.2%)	0.0145
Dialysis time (Month)	54.75 ± 50.97	39.88 ± 37.13	0.254

#### 3.1.1 Preoperative and postoperative laboratory parameter statistical analysis of ReTR and FTR group

**Figure 1:**
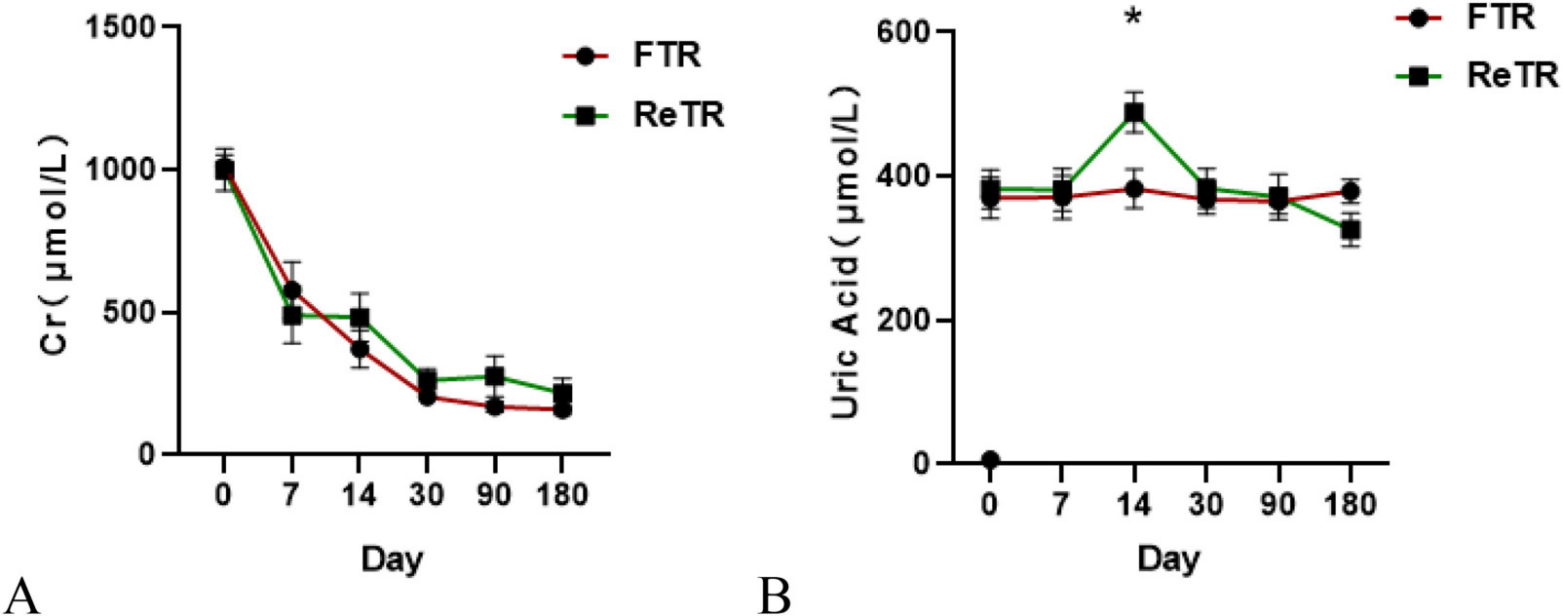
Paired line graphs compare changes in serum creatinine (panel **A**) and uric acid (panel **B**) over 180 days post-transplant between FTR (red line, circles) and ReTR (green line, squares) groups; creatinine levels decrease similarly in both groups, while uric acid peaks higher in ReTR at day 14 before normalizing, with a significant difference. *P < 0.05. FTR: First-time recipients, ReTR: re-transplantation recipients.

A comparison of clinical data between ReTR and FTR group revealed no significant differences in preoperative laboratory parameters between the two groups (Table 2). At 7 days postoperatively, the platelet count (145.22 ± 52.39 vs. 194.33 ± 63.82, *P* = 0.006) and serum albumin levels (34.1 ± 2.45 vs. 35.7 ± 2.86, *P* = 0.046) in the ReTR group were significantly lower than those in the FTR group (Table 3). Follow-up measurements of serum creatinine and serum uric acid, revealed that the ReTR group exhibited higher creatinine levels compared to the FTR group, though without statistical significance (*P* < 0.05) (see Table 3, Figure 1A). At 14 days postoperatively, the uric acid levels inReTR group were significantly higher than FTR group (488.82 ± 137.5 vs. 382.9 ± 132.75, *P* = 0.021) (see Figure 1B).

**Table 2:** Comparison of preoperative laboratory parameters between ReTR and FTR group.

Parameter	FTR group (*n* = 24)	ReTR group (*n* = 24)	*P* value
White blood cells (×10^9^/L)	7.6 ± 2.8	6.82 ± 1.74	0.255
Hemoglobin (g/L)	117.08 ± 16.82	116.29 ± 21.86	0.889
Platelet count (×10^9^/L)	220.25 ± 66.69	186.25 ± 57.69	0.065
Lymphocyte percentage (%)	17.55 ± 6.61	18.75 ± 8.35	0.586
Neutrophil percentage (%)	75.25 ± 9.45	72.55 ± 10.87	0.363
Low-density lipoprotein (mmol/L)	2.18 ± 0.76	2.18 ± 0.75	0.982
Triglycerides (mmol/L)	1.95 ± 1.1	3.17 ± 3.16	0.097
Blood glucose (mmol/L)	5.76 ± 1.26	6.35 ± 2	0.263
Serum albumin (g/L)	43.36 ± 7.6	45.08 ± 5.64	0.38
Sodium (mmol/L)	138.98 ± 3.21	138.81 ± 2.71	0.851
Potassium (mmol/L)	5.24 ± 0.73	4.87 ± 0.86	0.116
Calcium (mmol/L)	2.31 ± 0.21	2.37 ± 0.48	0.556
Phosphorus (mmol/L)	1.9 ± 0.54	1.84 ± 0.63	0.726
ALT (U/L)	11.77 ± 6.11	11.44 ± 7.31	0.868
AST (U/L)	13.53 ± 6.93	14.33 ± 6.93	0.693
AKP (U/L)	74 ± 17.13	92.54 ± 44.14	0.061
Creatinine (*μ*mol/L)	1,012.2 ± 198.29	1,000.49 ± 362.67	0.89
Urea (mmol/L)	22.27 ± 9.33	21.96 ± 9.38	0.909
Uric acid (μmol/L)	369.95 ± 141.39	382.15 ± 133.57	0.775
T cells (%)	70.61 ± 14.47	69.75 ± 14.96	0.849
Tc cells (%)	24.78 ± 6.26	30.3 ± 13.4	0.085
Th cells (%)	42.52 ± 12.38	36.65 ± 13.1	0.139
NK cells (%)	17.74 ± 10.06	19.95 ± 11.67	0.508
B cells (%)	9.7 ± 8.84	8.25 ± 5.93	0.539
Th/Ts [Th/Ts]	1.81 ± 0.72	1.43 ± 0.82	0.116

**Table 3:** Comparison of postoperative laboratory parameters between ReTR and FTR group.

Parameter	FTR group (*n* = 24)	ReTR group (*n* = 24)	*P* value
7d-White blood cells (×10^9^/L)	9.17 ± 2.54	7.83 ± 2.32	0.064
7d-Hemoglobin (g/L)	93.88 ± 12.01	88.74 ± 12.62	0.16
7d-Platelet count (×10^9^/L)	194.33 ± 63.82	145.22 ± 52.39	0.006
7d-Lymphocyte percentage (%)	7.19 ± 6.1	6.3 ± 4.04	0.561
7d-Neutrophil percentage (%)	83.63 ± 6.99	85.43 ± 6.07	0.352
7d-Serum albumin (g/L)	35.7 ± 2.86	34.1 ± 2.45	0.046
7d-Urea (mmol/L)	29.5 ± 13.23	29.75 ± 14.5	0.951
7d- T cells (%)	54.58 ± 19.32	57.2 ± 24.27	0.764
7d- Tc cells (%)	25.17 ± 9.18	29 ± 16.9	0.487
7d- Th cells (%)	27.5 ± 14.06	25.67 ± 13.01	0.731
7d-NK cells (%)	15.75 ± 13.69	9.33 ± 7.26	0.13
7d- B cells (%)	22.92 ± 12.97	28.87 ± 21.42	0.406
7d-Th/Ts [Th/Ts]	1.17 ± 0.69	1.08 ± 0.54	0.718

### 3.2 Comparison of prognosis between ReTR and FTR group

#### 3.2.1 Comparison of patient survival rates between ReTR and FTR group

**Figure 2:**
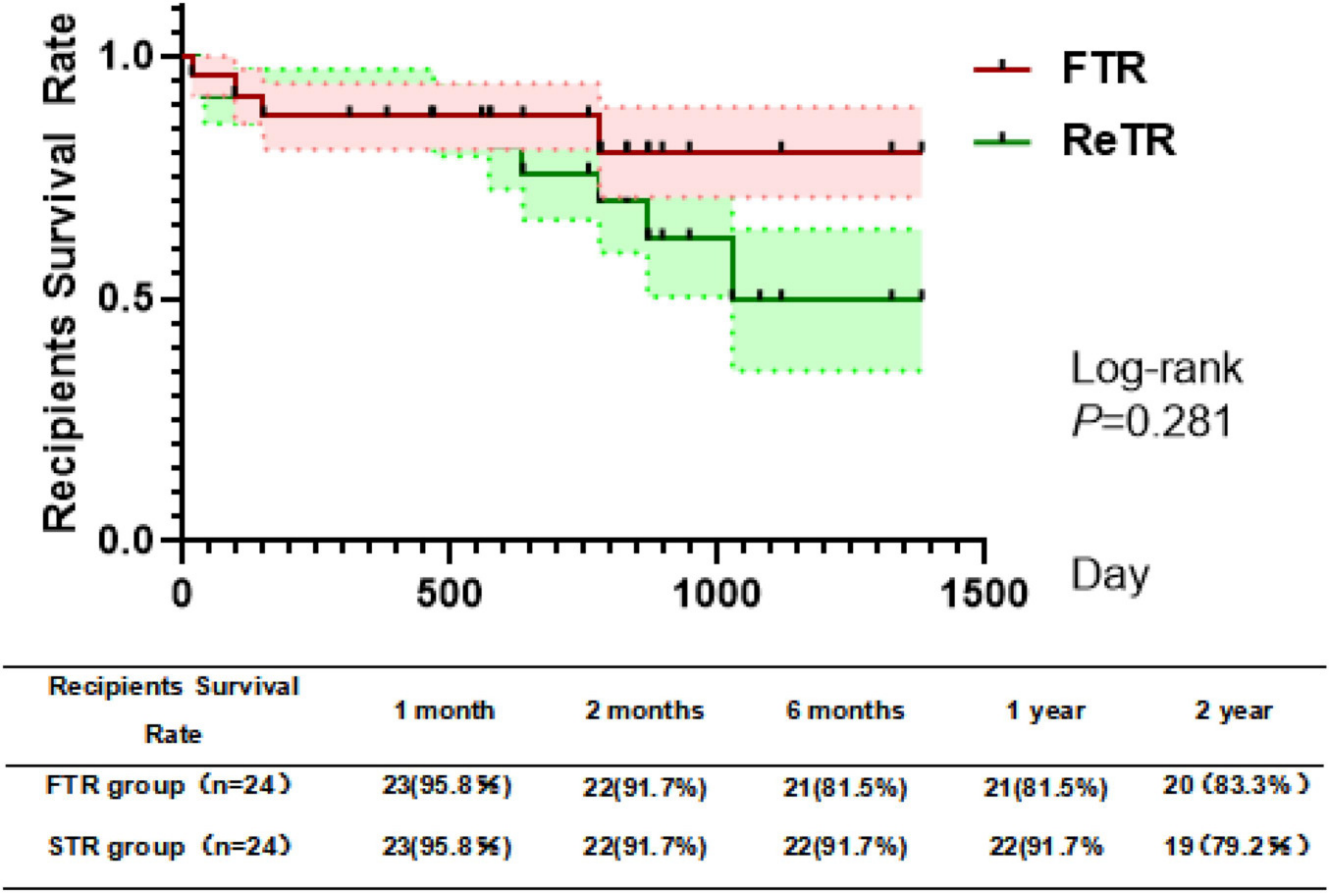
Kaplan-Meier survival curve compares recipients' survival rates over 1500 days for FTR (red) and ReTR (green) groups, with shaded confidence intervals and a logrank p-value of 0.281. Survival rates for each group at 1 month, 2 months, 6 months, 1 year, and 2 years are listed in the corresponding table below the graph. FTR: First-time recipients, ReTR: re-transplantation recipients.

The causes of death were postoperative infection, gastrointestinal bleeding, and intracerebral hemorrhage (see Table 4). The survival rates of the ReTR group were comparable to those of the FTR group at 1 and 2 months. At 6 months, the survival rate of the ReTR group declined, but the Log Rank test showed no significant difference between the two groups (*P* = 0.281) (see Figure 2). Based on univariate analysis of recipient survival outcomes, variables with *P*-values less than 0.1 and clinically relevant indicators including DGF, serum uric acid at 14 days postoperatively, platelet count at 7 days postoperatively, serum albumin at 7 days postoperatively, history of coronary heart disease, preoperative low-density lipoprotein, and secondary transplantation were included in multivariate Cox regression analysis. The results indicated that hypoalbuminemia at 7 days postoperatively was an independent risk factor for mortality in transplant recipients (HR: 2.387, 95.0% CI: 1.072–5.319, *P* = 0.033) (see Table 5).

**Figure 3:**
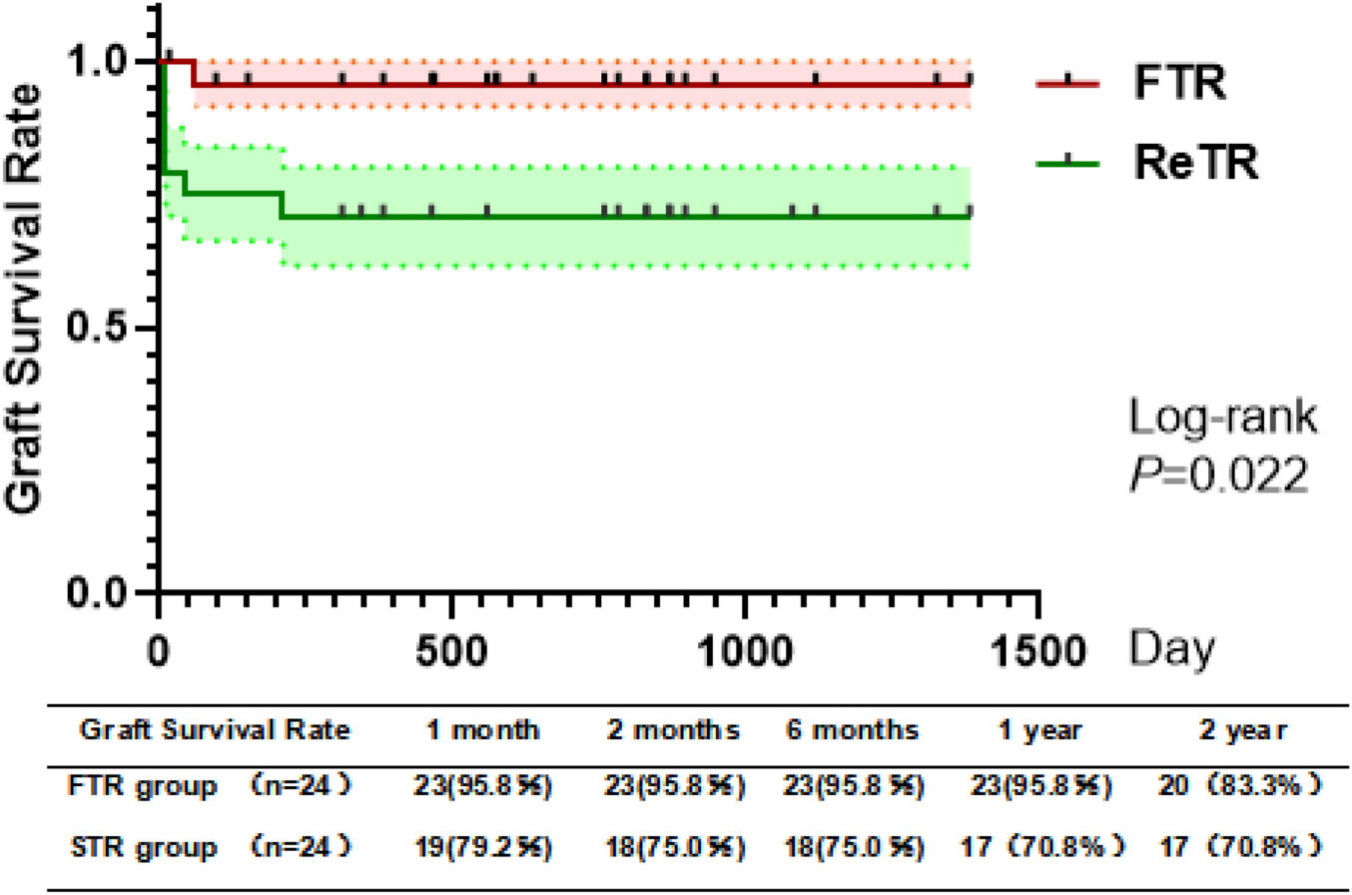
Kaplan-Meier line graph comparing graft survival rates over 1500 days for FTR (red line) and ReTR (green line) groups, showing higher survival for FTR; survival percentages at specific time points are listed in the table below, and a significant difference is indicated by log-rank (*P* = 0.022). FTR: First-time recipients, ReTR: re-transplantation recipients.

**Table 4:** Postoperative mortality and graft failure rates in renal transplant recipients.

Parameter	Diagnosis	ReTR	FTR
Causa mortis	Postoperative infection	2 (8.33%)	2 (8.33%)
	Hemorrhage of digestive tract	1 (4.17%)	1 (4.17%)
	Cerebral hemorrhage	1 (4.17%)	
Causes of transplant renal failure	Postoperative infection	2 (8.33%)	
	Acute rejection	1 (4.17%)	1 (4.17%)
	Chronic rejection	1 (4.17%)	
	Transplant renal artery stenosis	1 (4.17%)	
	Thrombosis of the iliac external vein and femoral vein	1 (4.17%)	
	Perirenal hematoma	1 (4.17%)	

**Table 5:** Multivariate Cox regression analysis of mortality in renal transplant recipients.

Parameter	*B*	SE	Wald	*P* value	HR	95.0% CI
DGF	1.785	1.685	1.121	0.29	5.957	0.219–161.964
14d-Uric acid (μmol/L)	0.019	0.01	3.679	0.055	1.019	1.001–1.039
Coronary heart disease	2.85	2.774	1.055	0.304	17.28	0.075–3,973.362
7d-Platelet (×10^9^/L)	−0.031	0.018	3.189	0.074	0.969	0.937–1.003
Low-density lipoprotein (mmol/L)	3.747	1.988	3.551	0.06	42.375	0.86–2,087.052
7d-Serum albumin (g/L)	−0.871	0.409	4.537	0.033	0.419	0.188–0.933
Renal re-transplantation	0.282	1.211	0.054	0.816	1.326	0.123–14.252

#### 3.2.2 Comparison of graft survival rates between ReTR and FTR group

The causes of graft failure included acute/chronic rejection reactions, perirenal hematoma, arterial stenosis of the transplanted kidney, and thrombosis of the external iliac vein and femoral vein (see Table 4). The survival rates of ReTR group grafts at 1, 2, 6, 12, and 24 months were significantly lower than those of FTR group, with a statistically significant difference between the two groups according to the Log Rank test (*P* = 0.022) (see Figure 3). Based on univariate analysis of recipient graft survival, variables with *P*-values less than 0.1 and clinically relevant indicators including secondary transplantation, DGF, serum uric acid at 14 days postoperatively, history of coronary heart disease, gender, systolic blood pressure, and diastolic blood pressure were included in multivariate Cox regression analysis. The results indicated that secondary transplantation (HR: 81.867, 95.0% CI: 1.164–5,758.747, *P* = 0.042) and postoperative DGF (HR: 156.163, 95.0% CI: 1.249–19,531.98, *P* = 0.04) were independent risk factors for graft failure (see Table 6).

**Table 6:** Multivariate COX regression analysis of transplant renal failure.

Parameter	*B*	SE	Wald	*P* value	HR	95.0% CI
Renal re-transplantation	4.405	2.17	4.12	0.042	81.867	1.164–5,758.747
DGF	5.051	2.464	4.203	0.04	156.163	1.249–19,531.98
14d-Uric acid (μmol/L)	0.007	0.007	0.92	0.337	1.007	0.993–1.02
Coronary heart disease	1.082	1.51	0.513	0.474	2.951	0.153–56.955
Male	0.589	1.066	0.305	0.581	1.802	0.223–14.563
SBP (mmHg)	0.005	0.079	0.004	0.948	1.005	0.86–1.175
DBP (mmHg)	−0.105	0.195	0.287	0.592	0.901	0.614–1.321

#### 3.2.3 Comparison of DGF incidence after transplantation

**Figure 4:**
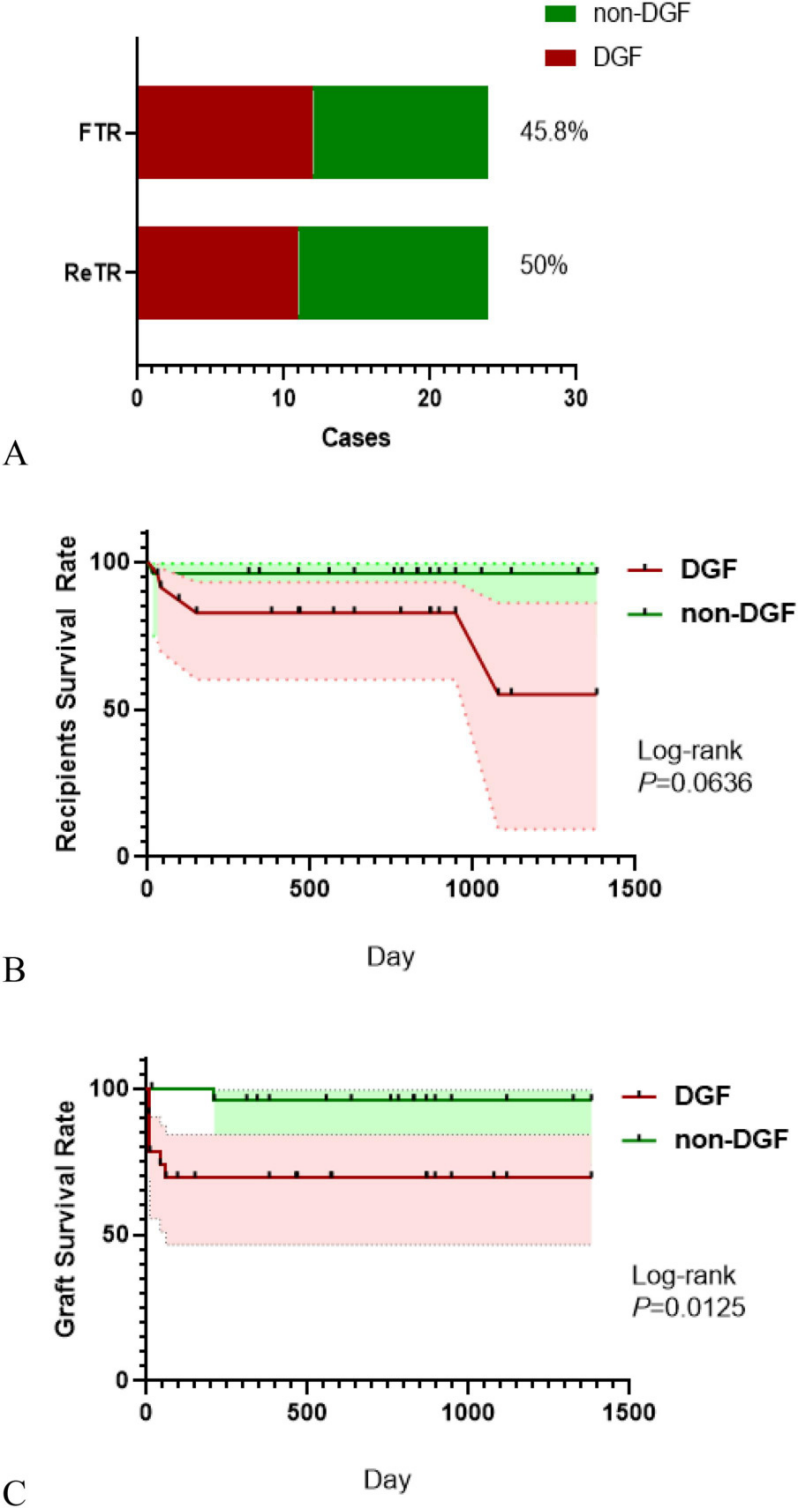
Figure contains three data visualizations. Panel **A** is a horizontal bar chart comparing the percentage of DGF and non-DGF cases in FTR and ReTR groups, showing 45.8% and 50% non-DGF, respectively. Panel **B** is a Kaplan-Meier curve showing higher overall recipient survival rates for non-DGF (green) compared to DGF (red) over 1,500 days with a log-rank *P*-value of 0.0636. Panel **C** is a Kaplan-Meier curve showing higher graft survival for non-DGF compared to DGF with a log-rank *P*-value of 0.0125,with a significant difference. DGF: delayed graft function, FTR: First-time recipients, ReTR: re-transplantation recipients.

DGF occurred in 11 patients in the FTR group and 12 patients in the ReTR group, with comparable incidence rates in both groups showing no statistically significant difference (45.8% vs 50%, *P* = 0.773). (See Figure 4A) Kaplan-Meier curve showed higher overall recipient survival rates for non-DGF group compared to DGF group but no significant difference (log-rank *P* = 0.0636). (See Figure 4B) Compared to DGF group, non-DGF group had higher graft survival with a significant difference (log-rank *P* = 0.0125) (see Figure 4C).

## 4 Discussion

The condition of recipients undergoing repeat kidney transplantation is more complex compared to the initial transplant. Factors such as vascular limitations, potential immune sensitization, preoperative medications, and disease status make the surgical procedure and treatment process more challenging (9). This study revealed differences in certain pre-and postoperative indicators between patients undergoing repeat kidney transplantation and those receiving first-time kidney transplantation from the same donors contralateral side. The findings included a higher proportion of PRA-positive patients in the repeat transplant group, lower platelet and serum albumin levels at 7 days postoperatively, and a significant increase in uric acid at 14 days postoperatively. Clinical outcomes indicated that the survival rate of transplanted kidneys in ReTR group was lower than that in FTR group, but there was no significant difference in DGF or overall survival rates. Hypoalbuminemia at 7 days postoperatively was an independent risk factor for mortality in patients after transplantation. Secondary transplantation and postoperative DGF were independent risk factors for graft failure.These results suggest that under strict screening and management, repeat transplantation can generally prolong the survival time and improve the quality of life for uremic patients, demonstrating its feasibility.

The immune status of patients undergoing repeat kidney transplantation is more complex, with an increased incidence of sensitization among repeat transplant recipients. This represents a major immunological barrier that must be overcome in transplant medicine. Elevated levels of population-reactive antibodies further complicate the matching of suitable donor kidneys (1). Our study revealed that patients in the preoperative repeat kidney transplantation group had a higher proportion of PRA positivity, which was associated with the induction of pre-existing antibodies against HLA antigens (sensitized state) during the first transplantation, thereby significantly increasing the risk of rejection after repeat kidney transplantation (10). Preoperative desensitization therapy can be performed through plasma exchange, immune adsorption, or the application of novel biologics (such as rituximab) (11).

To preserve residual renal function or avoid high-risk surgeries, the first transplant kidney was not resected, and a second kidney was transplanted into the contralateral iliac fossa. Patients often exhibit extensive intra-abdominal adhesions, particularly due to fibrosis of the surrounding tissues around the transplanted kidney, which leads to unclear anatomical structures and increases the risk of complications such as bleeding. In the study, the levels of platelets and serum albumin in the second transplant group were significantly lower than those in the first transplant group at 7 days postoperatively. The thrombocytopenia may be associated with the following factors: the second transplant procedure is generally more complex, with prolonged operative time and greater trauma, resulting in increased consumption of platelets at the wound site (12). Bone marrow suppression induced by immunosuppressive agents (e.g., anti-thymocyte globulin) (13) and the immune system reconstruction after retransplantation may be more complex, with the occurrence of antibody-mediated platelet destruction (14), leading to increased platelet phagocytosis. Hypoalbuminemia may result from the complexity of secondary surgery and the release of postoperative inflammatory factors, such as interleukin-6 (IL-6) and tumor necrosis factor-α (TNF-α), which inhibit hepatic albumin synthesis while increasing vascular permeability, leading to albumin leakage into the interstitial space (15, 16). The use of high-dose corticosteroids increases protein catabolism, further reducing albumin levels. Insufficient nutrient intake and increased metabolic demands often coexist with underlying conditions such as anemia and hypoalbuminemia in patients undergoing secondary transplantation. Postoperative metabolic demands or inadequate food intake may lead to insufficient protein synthesis (17). Additionally, due to incomplete recovery of the transplanted kidney function, if the glomerular filtration rate (GFR) is low, proteinuria or reabsorption impairment may occur, which could also exacerbate albumin loss.

The uric acid level in the retransplantation group was significantly higher than that in the first transplantation group at 14 days postoperatively. Due to the long-term use of immunosuppressants before surgery in retransplantation patients, the cumulative effect of these drugs is pronounced. Commonly used calcineurin inhibitors such as tacrolimus and cyclosporine can affect uric acid levels, and their nephrotoxicity may impair renal tubular function, thereby reducing the efficiency of uric acid excretion (18, 19). The transient decline in glomerular filtration rate (GFR) caused by DGF can directly reduce uric acid excretion (20). Long-term uremic environment and chronic graft injury from previous transplantation may predispose patients undergoing retransplantation to metabolic syndrome (21). Manifested as insulin resistance and obesity, both of which can collectively elevate serum uric acid levels by increasing uric acid production and reducing its excretion.

The postoperative graft survival period in patients undergoing repeat kidney transplantation is lower than that in first-time transplant recipients, and the incidence of postoperative complications is significantly higher in repeat transplant patients. This is associated with the increased surgical difficulty of the second procedure and the preoperative use of immunosuppressants such as calcineurin inhibitors (CNI) or glucocorticoids to prevent rejection. The nephrotoxicity of CNI agents like tacrolimus and cyclosporine may accelerate the loss of graft function. Additionally, the management of underlying conditions such as diabetes and hypertension further increases the complexity of the procedure (22). In our study, the incidence of DGF was comparable, which may be related to the control group being the contralateral kidney derived from the same donor. Donor factors play a significant role in the occurrence of DGF (23).

Although there were differences in postoperative platelet count, serum albumin levels, and serum uric acid levels between the two groups of patients, these parameters could be rapidly corrected with active treatment. The relationship between these changes and long-term survival of the transplanted kidney still requires further investigation. Although the survival rate of the transplanted kidney in patients undergoing repeat kidney transplantation was lower than that in first-time transplant recipients, the DGF (donor graft function) and overall survival rates were similar in both groups. Improving outcomes for patients undergoing secondary transplantation requires comprehensive interventions across multiple dimensions, including donor selection, organ preservation, recipient management, and perioperative care. Donors should undergo rigorous maintenance and evaluation; organ preservation and transportation protocols must be optimized to minimize cold ischemia and thermal ischemia durations; recipient comorbidities require active management, with particular attention to nutritional status, gastrointestinal function, and infection risks beyond cardiovascular and cerebrovascular conditions; perioperative management should ensure hemodynamic stability through rational fluid administration and optimized immunosuppressive regimens; for high-risk recipients, lymphocyte-depleting agents may be considered as induction therapy to reduce immune-mediated injury.

Limitations of this study: The control group consisted of first-time kidney transplant recipients from the same donors contralateral side, which helped exclude donor-related confounding factors. However, as a retrospective study, it still carries potential confounding bias. Due to logistical constraints, routine postoperative PRA testing was not performed. Additionally, the limited number of matched recipients for secondary transplants resulted in a small sample size, necessitating future multicenter studies with larger cohorts.

## Data Availability

The raw data supporting the conclusions of this article will be made available by the authors, without undue reservation.
